# Supplementary figures and images for: In situ mutational screening and CRISPR interference define apterous cis-regulatory inputs during compartment boundary formation
Source: eLife. 2026 May 22;12:RP91713. doi: 10.7554/eLife.91713 (PMC13197166; doi:10.7554/eLife.91713)

## Supplementary File 1.

OR463 sequence and sub-regions

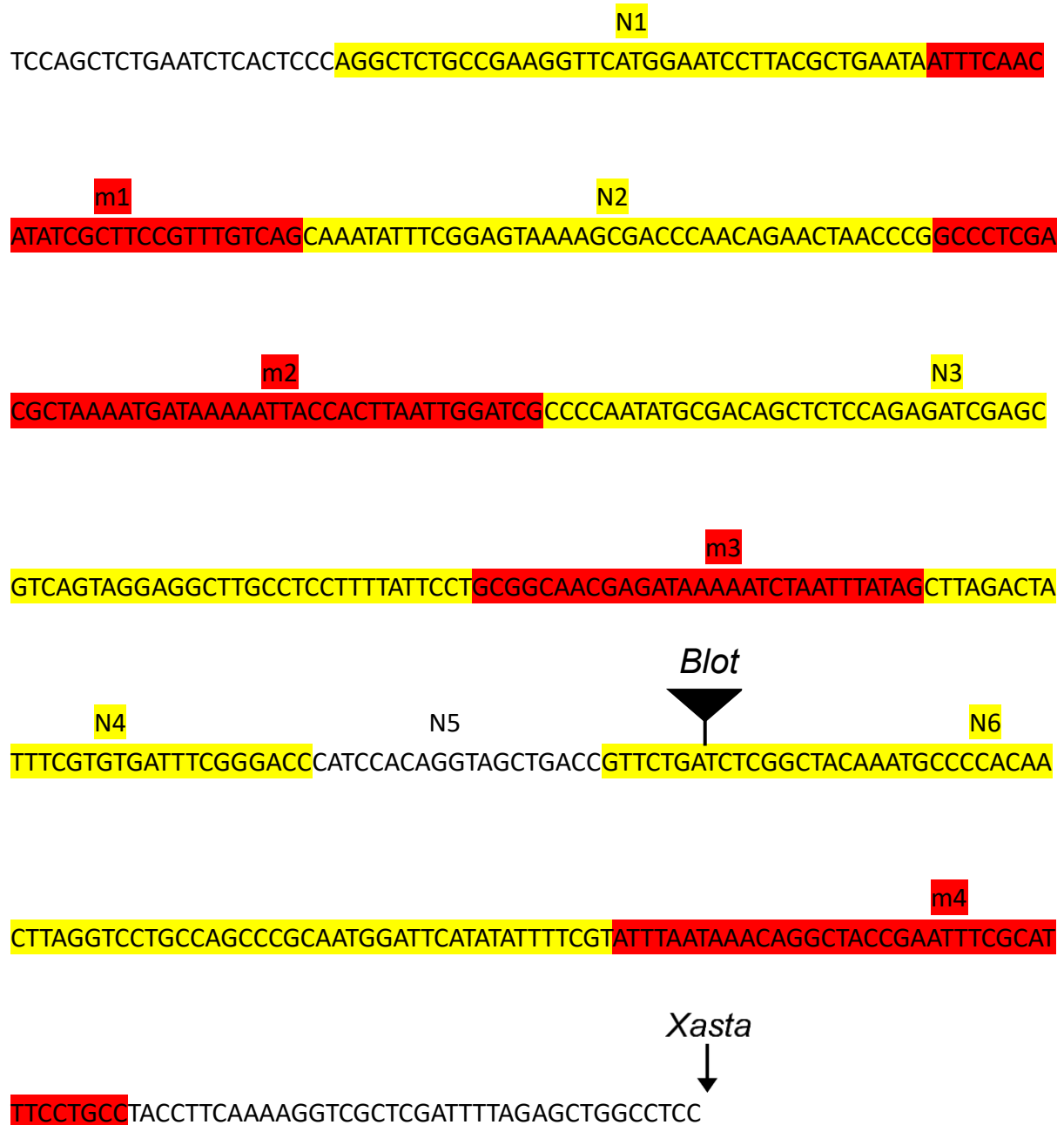

Supplement: Supplementary file 1. — Conserved sub-regions are highlighted in red (m1–m4; most conserved), whereas less conserved subregions are highlighted in yellow (N1–N6). The chromosomal breakpoint of the apXasta mutant and the insertion site of apBlot are also indicated. [file elife-91713-supp1.pdf]
