# Supplementary material for: In situ mutational screening and CRISPR interference define apterous cis-regulatory inputs during compartment boundary formation
Source: eLife. 2026 May 22;12:RP91713. doi: 10.7554/eLife.91713 (PMC13197166; doi:10.7554/eLife.91713)
Supplement: Supplementary file 2. [file elife-91713-supp2.docx]

| **Fly stock** | | **Plasmid used for its generation:** | | Stock used to inject the plasmids | |
| --- | --- | --- | --- | --- | --- |
| *apR2-WT* | | MS377 pRMCEentry –attB +yellow +WT in pUC57-Kan | | apOR463 landing site | |
| *apR2-Δm1* | | MS378 pRMCEentry –attB +yellow +m1 in pUC57-Kan | | apOR463 landing site | |
| *apR2-Δm2* | | MS379 pRMCEentry –attB +yellow +m2 in pUC57-Kan | | apOR463 landing site | |
| *apR2-Δm3* | | MS380 pRMCEentry –attB +yellow +m3 in pUC57-Kan | | apOR463 landing site | |
| *apR2-Δm4* | | DB341 pRMCEentry –attB +yellow +m4 in pUC57-Kan | | apOR463 landing site | |
| *apR2-Δm1m4* | | MS381 pRMCEentry –attB +yellow +m1m4 in pUC57-Kan | | apOR463 landing site | |
| *apR2-Δm1.1m4* | | MS383 pRMCEentry –attB +yellow +m1m4 in pUC57-Kan | | apOR463 landing site | |
| *apR2-Δm1.2m4* | | MS354 pRMCEentry –attB +yellow +m1.2m4 in pUC57-Kan | | apOR463 landing site | |
| *apR2-Δm1.3m4* | | MS384 pRMCEentry –attB +yellow +m1.3m4 in pUC57-Kan | | apOR463 landing site | |
| *apR2-Δm3.1* | | MS385 pRMCEentry –attB +yellow +m3.1 in pUC57-Kan | | apOR463 landing site | |
| *apR2-Δm3.2* | | MS386 pRMCEentry –attB +yellow +m3.2 in pUC57-Kan | | apOR463 landing site | |
| *apR2-Δm3.3* | | DB342 pRMCEentry –attB +yellow +m3.3 in pUC57-Kan | | apOR463 landing site | |
| *apR2-Δm3.4* | | MS387 pRMCEentry –attB +yellow +m3.4 in pUC57-Kan | | apOR463 landing site | |
| *apR2-ΔN1* | | MS388 pRMCEentry –attB +yellow +N1 in pUC57-Kan | | apOR463 landing site | |
| *apR2-ΔN2* | | MS389 pRMCEentry –attB +yellow +N2 in pUC57-Kan | | apOR463 landing site | |
| *apR2-ΔN3* | | MS390 pRMCEentry –attB +yellow +N3 in pUC57-Kan | | apOR463 landing site | |
| *apR2-ΔN4* | | MS391 pRMCEentry –attB +yellow +N4 in pUC57-Kan | | apOR463 landing site | |
| *apR2-ΔN6* | | MS392 pRMCEentry –attB +yellow +N5 in pUC57-Kan | | apOR463 landing site | |
| *apR2-Δm1m2m4* | | MS396 pRMCEentry –attB +yellow +m1m2m4 in pUC57-Kan apOR463 landing site | | | |
| *R2-OR463m3ΔAA* | | pRMCEentry –attB +yellow +m3ΔAA in pUC57-Kan apOR463 landing site | | | |
| *R2-OR463m3+6bp* | | pRMCEentry –attB +yellow +m3+6bp in pUC57-Kan apOR463 landing site | | | |
| *R2-OR463m2Δ6bpm3* | | pRMCEentry –attB +yellow +m2Δ6bpm3 in pUC57-Kan apOR463 landing site | | | |
| *apΔN5* | | Isolated as an indel at one of the gRNAs used to stablish the apR2 landing site | | | |
| m3 substitution library | |  | |  | |
| *R2-m3.1-A* | | pRMCEentry –attB +yellow +m3.1-A in pUC57-Kan | | apOR463 landing site | |
| *R2-m3.1-B* | | pRMCEentry –attB +yellow +m3.1-B in pUC57-Kan | | apOR463 landing site | |
| *R2-m3.1-C* | | pRMCEentry –attB +yellow +m3.1-C in pUC57-Kan | | apOR463 landing site | |
| *R2-m3.1-D* | | pRMCEentry –attB +yellow +m3.1-D in pUC57-Kan | | apOR463 landing site | |
| *R2-m3.1-E* | | pRMCEentry –attB +yellow +m3.1-E in pUC57-Kan | | apOR463 landing site | |
| *R2-m3.1-F* | | pRMCEentry –attB +yellow +m3.1-F in pUC57-Kan | | apOR463 landing site | |
| *R2-m3.1-G* | | pRMCEentry –attB +yellow +m3.1-G in pUC57-Kan | | apOR463 landing site | |
| *R2-m3.1-H* | | pRMCEentry –attB +yellow +m3.1-H in pUC57-Kan | | apOR463 landing site | |
| *R2-m3.1-I* | | pRMCEentry –attB +yellow +m3.1-I in pUC57-Kan | | apOR463 landing site | |
| *R2-m3.1-J* | | pRMCEentry –attB +yellow +m3.1-J in pUC57-Kan | | apOR463 landing site | |
| *R2-m3.1-K* | | pRMCEentry –attB +yellow +m3.1-K in pUC57-Kan | | apOR463 landing site | |
| *R2-m3.1-L* | | pRMCEentry –attB +yellow +m3.1-L in pUC57-Kan | | apOR463 landing site | |
| *R2-m3.1-M* | | pRMCEentry –attB +yellow +m3.1-M in pUC57-Kan | | apOR463 landing site | |
| *R2-m3.1-N* | | pRMCEentry –attB +yellow +m3.1-N in pUC57-Kan | | apOR463 landing site | |
| *R2-m3.1-O* | | pRMCEentry –attB +yellow +m3.1-O in pUC57-Kan | | apOR463 landing site | |
| *R2-m3.1-P* | | pRMCEentry –attB +yellow +m3.1-P in pUC57-Kan | | apOR463 landing site | |
| *R2-m3.1-Q* | | pRMCEentry –attB +yellow +m3.1-Q in pUC57-Kan | | apOR463 landing site | |
| *R2-m3.1-R* | | pRMCEentry –attB +yellow +m3.1-R in pUC57-Kan | | apOR463 landing site | |
| *R2-m3.1-S* | | pRMCEentry –attB +yellow +m3.1-S in pUC57-Kan | | apOR463 landing site | |
| *R2-m3.1-T* | | pRMCEentry –attB +yellow +m3.1-T in pUC57-Kan | | apOR463 landing site | |
| *R2-m3.1-U* | | pRMCEentry –attB +yellow +m3.1-U in pUC57-Kan | | apOR463 landing site | |
| Other lines | |  | |  | |
| *apEΔm3-LacZ* | attB-apEΔm3-LacZ | | (attP-86Fb)ZH | |  |
| *UAS-dCas9* | pUAStattB-dCas9 | | (attP-86Fb)ZH | |  |
| *U6-OR463.gRNAx4* | pCFD5-U6-OR463.gRNAx4 | | attp40 (BL25709) | |  |
| *U6-antp.gRNAx3* | pCFD5-U6-antp.gRNAx3 | | attp40 (BL25709) | |  |
| *CON2-mKate2* | CON2-mKate2 | | (attP-86Fb)ZH | |  |
| *CON5-eGFP-NLS* | CON5-eGFP-NLS | | (attP-86Fb)ZH | |  |
