# Supplementary material for: In situ mutational screening and CRISPR interference define apterous cis-regulatory inputs during compartment boundary formation
Source: eLife. 2026 May 22;12:RP91713. doi: 10.7554/eLife.91713 (PMC13197166; doi:10.7554/eLife.91713)
Supplement: Supplementary file 3. [file elife-91713-supp3.docx]

| **Primer name** | | **Sequence 5'-3'** | | **Plasmid generated with it** | |
| --- | --- | --- | --- | --- | --- |
| m1.1 (A317) | gttcatggaatccttacgctgaatatcgcttccgtttgtcagcaaatatttcgg | | MS383 pRMCEentry –attB +yellow +m1m4 in pUC57-Kan | |  |
| m1.2 (A318) | acgctgaataatttcaacatatgtcagcaaatatttcggagtaaaagc | | MS354 pRMCEentry –attB +yellow +m1.2m4 in pUC57-Kan | |  |
| m1.3 (A319) | tttcaacatatcgcttccgttcaaatatttcggagtaaaagcgac | | MS384 pRMCEentry –attB +yellow +m1.3m4 in pUC57-Kan | |  |
| m3.1 (A320) | cctccttttattcctgcggcaaccttagactatttcgtgtgatttcgg | | MS385 pRMCEentry –attB +yellow +m3.1 in pUC57-Kan | |  |
| m3.2 (A321) | ctccttttattcctgcggcaacaatctaatttatagcttagactatttcg | | MS386 pRMCEentry –attB +yellow +m3.2 in pUC57-Kan | |  |
| m3.3 (A322) | tttattcctgcggcaacgagataaaatagcttagactatttcgtgtg | | DB342 pRMCEentry –attB +yellow +m3.3 in pUC57-Kan | |  |
| m3.4 (A323) | gcaacgagataaaaatctaatttcttagactatttcgtgtgatttcgg | | MS387 pRMCEentry –attB +yellow +m3.4 in pUC57-Kan | |  |
| m1 Mutagenic Primer (A267) | gttcatggaatccttacgctgaatacaaatatttcggagtaaaagcgac | | MS378 pRMCEentry –attB +yellow +m1 in pUC57-Kan & MS381 (m1m4) | |  |
| m2 Mutagenic Primer (A268) | aagcgacccaacagaactaacccgccccaatatgcgacagctc | | MS379 pRMCEentry –attB +yellow +m2 in pUC57-Kan | |  |
| m3 Mutagenic Primer (A269) | gcttgcctccttttattcctcttagactatttcgtgtgatttcgg | | MS380 pRMCEentry –attB +yellow +m3 in pUC57-Kan | |  |
| m4 Mutagenic Primer (A270) | cgcaatggattcatatattttcgttaccttcaaaaggtcgctcg | | DB341 pRMCEentry –attB +yellow +m4 in pUC57-Kan & MS381 (m1m4) | |  |
| N1 mutagenetic primer (P43) | tgaatctcactcccatttcaacatatcgc | | MS388 pRMCEentry –attB +yellow +N1 in pUC57-Kan | |  |
| N2 mutagenetic primer (P44) | cttccgtttgtcaggccctcgacgctaaaatg | | MS389 pRMCEentry –attB +yellow +N2 in pUC57-Kan | |  |
| N3 mutagenetic primer (P45) | acttaattggatcggcggcaacgagataaaaatc | | MS390 pRMCEentry –attB +yellow +N3 in pUC57-Kan | |  |
| N4 mutagenetic primer (P46) | aatctaatttatagcatccacaggtagc | | MS391 pRMCEentry –attB +yellow +N4 in pUC57-Kan | |  |
| N6 mutagenetic primer (P47) | acaggtagctgaccatttaataaacagg | | MS392 pRMCEentry –attB +yellow +N5 in pUC57-Kan | |  |
